# Long Non-coding RNA FEZF1-AS1 Promotes Growth and Reduces Apoptosis Through Regulation of miR-363-3p/PAX6 Axis in Retinoblastoma

**DOI:** 10.1007/s10528-020-10026-7

**Published:** 2021-01-11

**Authors:** Xiuming Liu, Xiaofeng Li, Jianchang Li

**Affiliations:** Department of Ophthalmology, The Affiliated Huaian No. 1 People’s Hospital of Nanjing Medical University, No. 1, Huanghe Road, Huaiyin District, Huaian, 223300 Jiangsu China

**Keywords:** Retinoblastoma, FEZF1-AS1, miR-363-3p, PAX6, Cell growth

## Abstract

Retinoblastoma is the most common malignancy in children's eyes with high incidence. Long non-coding RNAs (lncRNAs) play important roles in the progression of retinoblastoma. LncRNA FEZF1 antisense RNA 1 (FEZF1-AS1) has been found to stimulate retinoblastoma. However, the mechanism of FEZF1-AS1 underlying progression of retinoblastoma is still unclear. In current study, FEZF1-AS1 was up-regulated in retinoblastoma tissues and cells. FEZF1-AS1 overexpression enhanced retinoblastoma cell viability, promoted cell cycle, and inhibited apoptosis. Conversely, FEZF1-AS1 knockdown reduced cell viability, cycle, and elevated apoptosis. The interaction between FEZF1-AS1 and microRNA-363-3p (miR-363-3p) was confirmed. FEZF1-AS1 down-regulated miR-363-3p and up-regulated PAX6. PAX6 was a target gene of miR-363-3p. EZF1-AS1 promoted retinoblastoma cell viability and suppressed apoptosis via PAX6. Further, we demonstrated that FEZF1-AS1 contribute to tumor formation in vivo. In conclusion, FEZF1-AS1 elevated growth and inhibited apoptosis by regulating miR-363-3p/PAX6 in retinoblastoma, which provide a new target for retinoblastoma treatment.

## Introduction

Retinoblastoma is the most common malignancy in children's eyes (Stark 2016). Retinoblastoma is accompanied by multiple lesions and easily exhibits intracranial and systemic metastasis when the tumor grows and breaks through the eyeball (Garsed et al. 2018). About 8000 cases being diagnosed yearly worldwide (Pascual-Pasto et al. 2019). There are 3540 new estimated cases occurring in the United States and approximately 1000 new cases happening each year in China (Chen et al. 2007; Siegel et al. 2018). Some remedies have been applied into treatment of patients with retinoblastoma, including chemotherapy combined with focal therapy (Ortiz and Dunkel 2016). However, the survival rate from patients with retinoblastoma is still unsatisfactory in the developing nations (Li et al. 2018b). Thus, it is interesting to search the useful biomarker for retinoblastoma treatment.

Long non-coding RNAs (lncRNAs) are more than 200 nt in length and function in limit of protein-coding potential (Zhu et al. 2018). It has reported that lncRNAs show important roles in the progression of human tumors (Tsai et al. 2011; Tang et al. 2013), including retinoblastoma (Wang et al. 2018; Yang and Peng 2018). LncRNA FEZF1 antisense RNA 1 (FEZF1-AS1), as one of lncRNAs, produces a 2564 bp transcript and is localized chromosome 7q31.32. Accumulating evidences have revealed that FEZF1-AS1 was abnormal expressed in tumor tissues and cells, and showed oncogenic effects on liver cancer (Gong et al. 2018), ovarian cancer (Zhao et al. 2018), and osteosarcoma (Zhou et al. 2018). Interestingly, FEZF1-AS1 was demonstrated to promote proliferation, migration, and invasion in retinoblastoma (Quan and Wang 2019). However, the mechanism of FEZF1-AS1 underlying development of retinoblastoma is still unclear.

MicroRNAs (miRNAs), 20–24 nucleotide, are an abundant class of small and highly-conserved endogenous non-coding RNA molecules (Bartel 2004). MiRNAs could bind to 3′-untranslated regions (UTR) of target mRNA and regulate different physiological processes (Sethi et al. 2013; Gu and Kay 2010). Accumulating evidences have shown that mRNAs played an important role in development of retinoblastoma (Reis et al. 2012). Notably, miR-363-3p has been found to take part in regulation of retinoblastoma (Ma et al. 2020). Thus, it is interesting to investigate the role of miR-363-3p in development of retinoblastoma.

In this study, the expression of FEZF1-AS1 was measured in retinoblastoma tissues and cells. The role of FEZF1-AS1 in progression of retinoblastoma was explored. Then, the interaction between FEZF1-AS1 and miR-363-3p was identified. We further investigated whether FEZF1-AS1 functions in progression of retinoblastoma through miR-363-3p. The study suggests that FEZF1-AS1 may provide a candidate target for retinoblastoma treatment.

## Materials and Methods

### Clinical Samples

The retinoblastoma specimens from 45 patients and 36 normal retinas were collected from The Affiliated Huaian No. 1 People’s Hospital of Nanjing Medical University. The research was carried out according to the World Medical Association Declaration of Helsinki and the Ethics Committee of The Affiliated Huaian No. 1 People’s Hospital of Nanjing Medical University. No subjects received preoperative radiotherapy or chemotherapy. Written informed consent was harvested from all subjects.

### Plasmid Construction

The pcDNA-FEZF1-AS1 expression plasmid (FEZF1-AS1) was constructed through pcDNA3.1 for endogenous expression. The FEZF1-AS1 sequence was synthesized and inserted into pcDNA3.1 between *EcoRI* and *XhoI*. The lnc-FEZF1-AS1 interference plasmid (shFEZF1-AS1) and control interference plasmid (shNC) were established via GenePharma (Shanghai, China). In addition, the open reading fragment sequence of PAX6 was subjected into PCR amplification from human genomic DNA and then cloned into pcDNA3.1 to generate the pcDNA3.1-PAX6 vector (PAX6).

### Cell Culture and Transfection

The human retinoblastoma cell lines (WERI-RB1, Y79) and normal retinal pigmented epithelium cell line (ARPE-19) were obtained from ATCC (Manassas, VA, USA). They were cultured in RPMI-1640 medium containing 10% fetal bovine serum (Beyotime, Shanghai, China) at 37 °C with 5% CO_2_. To overexpress FEZF1-AS1, WERI-RB1 cells were transfected with FEZF1-AS1. To knockdown FEZF1-AS1 expression, Y79 cells were transfected with shFEZF1-AS1 (F: 5′-AAACAUGGCAGCUACAAGACGGGUC-3′; R: 5′-GACCCGUCUUGUAGCUGCCAUGUUU-3′). Additionally, WERI-RB1 cells were co-transfected with FEZF1-AS1 and miR-363-3p mimic (5′-AAUUGCACGGUAUCCAUCUGUA-3′). Y79 cells were co-transfected with shFEZF1-AS1 and miR-363-3p inhibitor (5′-UUAACGUGCCAUAGGUAGACAU-3′). Besides, Y79 cells were co-transfected with shFEZF1-AS1 and PAX6. After transfection for 48 h, the cells were used to measurement.

### Quantitative PCR (qPCR)

Total RNAs were isolated through Trizol reagent. The concentration of total RNA was detected and reverse-transcribed via RT Primer Mix (Beyotime, Shanghai, China). ABI 7500 system was used for qPCR. The levels of FEZF1-AS1 and miR-363-3p were assessed and calculated by the 2^−ΔΔ*CT*^ method. The normalization was conducted with reference to expression of GAPDH and U6. The specific primers were as follows: FEZF1-AS1-F: 5′-AGAGGCTATGACTCAGGGTT-3′; FEZF1-AS1-R: 5′-TGTTGCTCCACAGTAAAGGT-3′; miR-363-3p-F: 5′-GCCGAGAATTGCACGGTAT-3′; miR-363-3p-R: 5′-CTCAACTGGTGTCGTGGA-3′; GAPDH-F: 5′-GCACCGTCAAGGCTGAGAAC-3′; GAPDH-R: 5′-TGGTGAAGACGCCAGTGG-3′; U6-F: 5′-CGCTTCGGCAGCACATATAC-3′; U6-R: 5′-AAATATGGAACGCTTCACGA-3’.

### Cell Proliferation Assay

Cells (2 × 10^3^) were seeded in 96-well plates. Then, the cells were incubated for 24, 48, and 72 h. At different time points, 10 μl Cell Counting Kit-8 (CCK-8) reagents were added into plates. After incubation for 4 h, absorbance was examined at 450 nm and cell viability was assessed.

### Cell Cycle Measurement

Cells (1 × 10^6^) were collected and fixed with ice-cold 70% ethanol overnight at 4 °C. The cells were washed thrice with PBS. Then, propidium iodide (PI) (Takara, Dalian, China) was used to incubate cells for 30 min at 37 °C. Finally, the cells were detected using flow cytometry.

### Cell Apoptosis Detection

Cells (3 × 10^5^) were harvested and resuspended with Annexin V-FITC/PI binding buffer. Subsequently, cells were stained with 5 μl Annexin V-FITC and 5 μl PI at 37 °C. Following 20 min of incubation in dark, cells were analyzed via flow cytometry. Data were calculated through FACS Diva software.

### Luciferase Reporter Assay

The wild-type (wt) or mut-type (mut) sequences of FEZF1-AS1 and PAX6 3′-UTR were obtained and cloned into pmirGLO vectors to generate the luciferase vectors, named by FEZF1-AS1-wt, FEZF1-AS1-mut, PAX6-wt, and PAX6-mut. Then, the WERI-RB1 was co-transfected with luciferase vectors and miR-363-3p inhibitor or negative control inhibitor (NC inhibitor) for 48 h. Y79 was co-transfected with luciferase vectors and miR-363-3p mimic or NC mimic for 48 h. A fluorescence microplate reader measured luciferase activity through Dual-Luciferase Reporter System Kit (Beyotime, Shanghai, China).

### RNA Immunoprecipitation (RIP) Assay

FEZF1-AS1overexpression was carried out in WERI-RB1 and Y79 cells. RIP lysis buffer was used to lysed cells and RIP assay was conducted via RIP RNA-Binding Protein Immunoprecipitation Kit (Abcam, Shanghai, China). Reaction system included Ago2 and IgG antibodies for RIP assays. The enrichments of FEZF1-AS1and miR-363-3p were identified using qPCR.

### Western Blot Analysis

Protein was extracted via reagent RIPA (Takara, Dalian, China). A BCA kit was used to detect protein concentration. Then, protein extracts (30–40 µg) were separated through SDS-PAGE and electroblotted onto PVDF membrane (EMD Millipore, Billerica, MA, USA). The membrane was covered with specific antibodies (all from Abcam, Cambridge, MA, USA) at 4 °C overnight. The specific antibodies included PAX6 (1:1000, ab195045), CyclinD1 (1:200, ab16663), p21 (1:2000, ab109520), Bcl-2 (1:1000, ab182858), Cleaved caspase-3 (1:500, ab32042), and GAPDH (1:5000, ab8245). Then, the membrane was incubated with secondary antibody for 50 min at 37 °C with a dilution of 1:3000. The proteins were examined through ECL reagent (Thermo Fisher Scientific, Waltham, MA, USA) and calculated using ImageJ software. A loading control was GAPDH.

### Immunohistochemistry

Tissues were fixed with 4% paraformaldehyde and 5-µm sections were harvested. The sections were deparaffinized and rehydrated and then blocked with goat serum for 10 min. Primary antibodies, including Ki67 and PAX6 (all from Abcam, Shanghai, China), were used to incubate the sections for 3 h at 37 °C. The secondary antibody covered the sections for 60 min at 37 °C. Nuclei were stained using DAPI. The photographs were acquired under a microscope.

### Tumor Xenograft Experiment

The 4-week-old male BALB/c nude mice were obtained and housed in a specific pathogen-free (SPF) room. Y79 cells (1 × 10^6^) expressing shFEZF1-AS1 were injected subcutaneously into the right fore-flank of nude mice. Size of the tumor was measured every week, and the weight of tumor was recorded. At 28 days post-injection, mice were euthanized and the tumors were excised. The volume was calculated through the following formula: *V* (mm^3^) = (length × width^2^)/2. All studies were approved by the Guide for the Care and Use of Laboratory Animals and the Ethics Committee of The Affiliated Huaian No. 1 People’s Hospital of Nanjing Medical University.

### Statistical Analysis

Data were presented as mean ± *SD*. Statistical analysis was conducted via GraphPad Prism software 5.0. Student’s *t* test and one-way analysis of variance (ANOVA) were used to analyze the data. The FEZF1-AS1 expression in relation to survival was assessed via the Kaplan–Meier analysis and the log-rank test. A *p* < 0.05 was considered significant.

## Results

### FEZF1-AS1 Was Increased in Retinoblastoma Patients and Cells

**Fig. 1:**
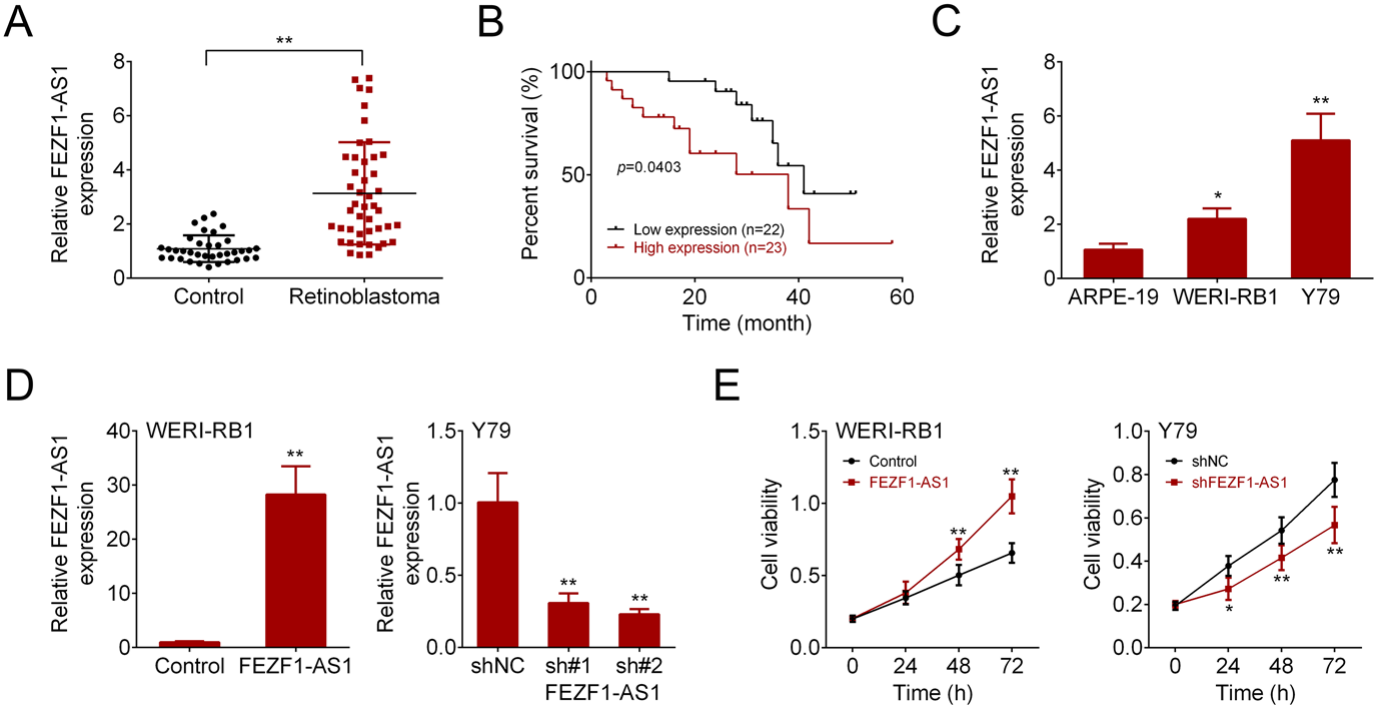
FEZF1-AS1 was increased in retinoblastoma patients and cells. **a** FEZF1-AS1 expression was detected via qRT-PCR in retinoblastoma patients. *n* = 45. **b** Kaplan–Meier analysis for retinoblastoma patients. *n* = 45. **c**, **d** The qRT-PCR analysis of FEZF1-AS1 expression in retinoblastoma cells. *n* = 3. **e** Cell viability was examined using CCK-8 assay. *n* = 3. *p < 0.05. **p < 0.01

We detected the FEZF1-AS1 expression in retinoblastoma patients and cells. The results of qRT-PCR showed that FEZF1-AS1 level was enhanced in retinoblastoma patients (Fig. 1a). Kaplan–Meier analysis revealed that retinoblastoma patients with high FEZF1-AS1 level had less survival compared with those with low FEZF1-AS1 level (Fig. 1b). Additionally, the FEZF1-AS1 level was examined in normal retinal pigmented epithelium cells (ARPE-19) and retinoblastoma cells (WERI-RB1 and Y79) using qRT-PCR. The results showed that FEZF1-AS1 level was increased in WERI-RB1 and Y79 (Fig. 1c). Further, the FEZF1-AS1 overexpression and FEZF1-AS1 silencing were successfully performed in the WERI-RB1 and Y79, respectively (Fig. 1d). CCK-8 assay demonstrated that FEZF1-AS1 overexpression promoted cell viability. Conversely, FEZF1-AS1 silencing inhibited cell viability (Fig. 1e). These data indicated that FEZF1-AS1 was abnormal expressed in retinoblastoma patients and cells, and FEZF1-AS1 affected retinoblastoma cell viability.

### FEZF1-AS1 Silencing Induced Retinoblastoma Cell Cycle Attest and Apoptosis

**Fig. 2:**
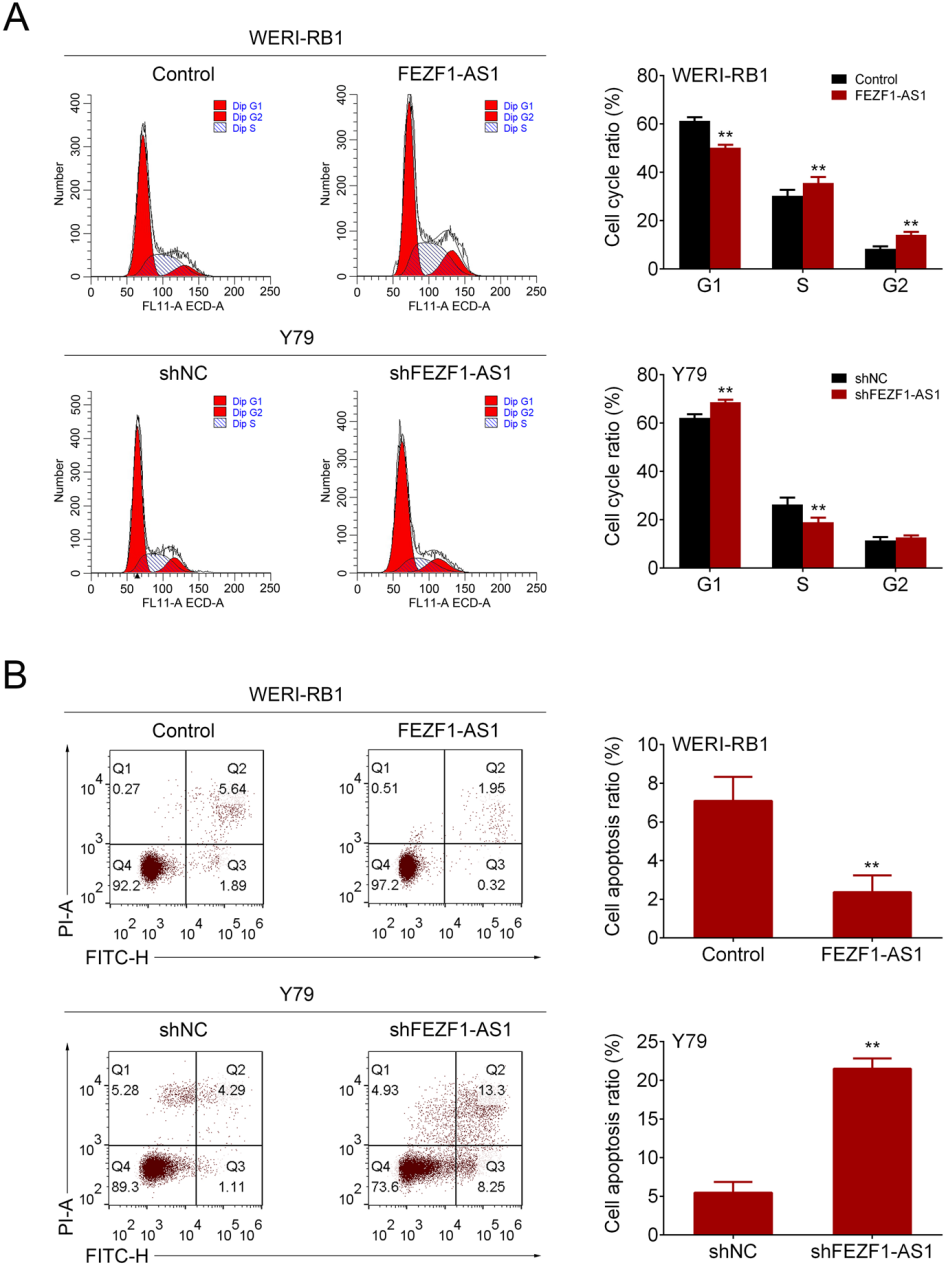
FEZF1-AS1 silencing induced retinoblastoma cell cycle attest and apoptosis. **a** Flow cytometry analysis detected cell cycle. **b** Cell apoptosis was measured through flow cytometry. *n* = 3. **p < 0.01

To explore the effect of FEZF1-AS1 on retinoblastoma cell cycle and apoptosis, the flow cytometry analysis was conducted. The results proved that FEZF1-AS1 overexpression reduced the cell population in the G1 phase and elevated cell population in the S phase. Conversely, FEZF1-AS1 silencing showed an opposite effect on retinoblastoma cell cycle (Fig. 2a). Further, flow cytometry analysis was performed and the results demonstrated that FEZF1-AS1 overexpression decreased cell apoptosis and FEZF1-AS1 silencing increased cell apoptosis (Fig. 2b). These findings implied that FEZF1-AS1 silencing induced retinoblastoma cell cycle attest and apoptosis.

### FEZF1-AS1 Was Confirmed to Sponge miR-363-3p

**Fig. 3:**
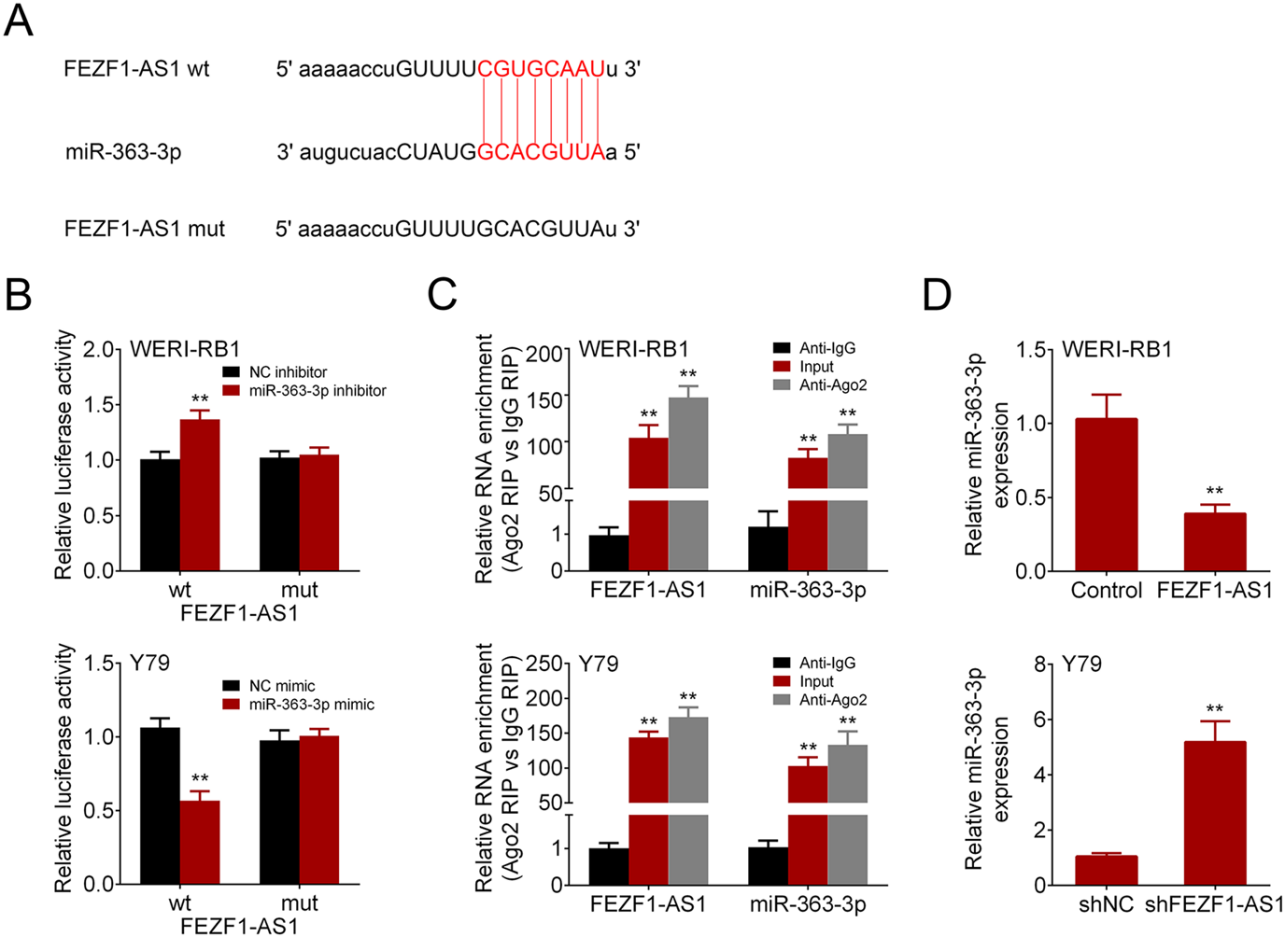
FEZF1-AS1 was confirmed to sponge miR-363-3p. **a** Starbase predicted that miR-363-3p could bind to FEZF1-AS1. **b** The interaction between FEZF1-AS1 and miR-363-3p was identified via luciferase reporter assay. **c** RIP assay demonstrated the relationship between AFAP1-AS1 and miR-497-5p. **d** MiR-363-3p expression was analyzed with qRT-PCR in retinoblastoma cells. *n* = 3. **p < 0.01

Starbase (http://starbase.sysu.edu.cn/index.php) predicted that miR-363-3p could bind to FEZF1-AS1 (Fig. 3a). Luciferase reporter assay demonstrated that miR-363-3p promoted luciferase activity of FEZF1-AS1-wt in WERI-RB1 and inhibited luciferase activity of FEZF1-AS1-wt Y79, whereas there was no influence on the luciferase activity of FEZF1-AS1-mut (Fig. 3b). The interaction between FEZF1-AS1 and miR-363-3p was further tested using RIP assay, and the results revealed that both FEZF1-AS1 and miR-363-3p were enriched in input or Ago2-containg immunoprecipitates compared with that in IgG immunoprecipitates (Fig. 3c). In addition, qRT-PCR analysis presented that FEZF1-AS1 overexpression repressed miR-363-3p level and FEZF1-AS1 silencing elevated miR-363-3p level (Fig. 3d). These findings suggested that FEZF1-AS1 could sponge miR-363-3p, and FEZF1-AS1 inhibited miR-363-3p level in retinoblastoma cells.

### FEZF1-AS1 Affected miR-363-3p/PAX6 Expression

**Fig. 4:**
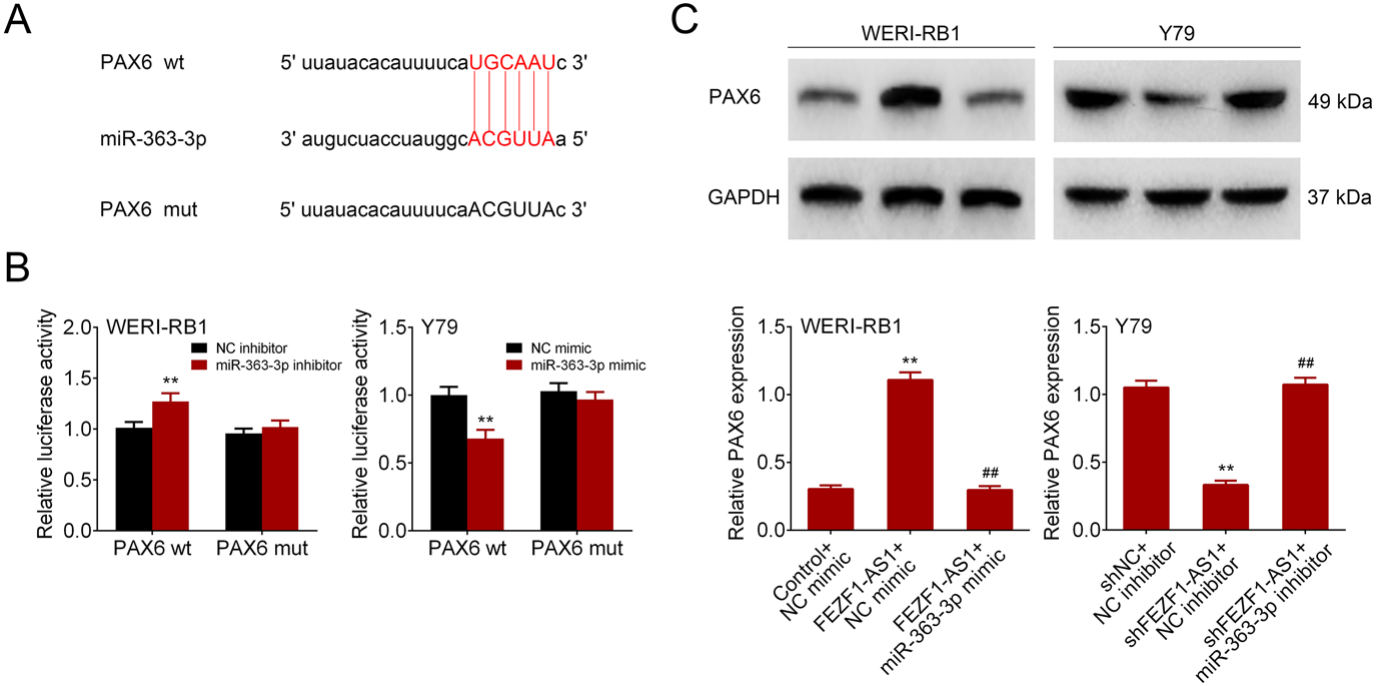
FEZF1-AS1 affected miR-363-3p/PAX6 expression. **a** Targetscan predicted that PAX6 could be targeted by miR-363-3p. **b** Luciferase reporter assay was used to prove the interaction between miR-363-3p and PAX6. *n* = 3. **p < 0.01. **c** PAX6 protein level was measured via Western blot analysis. *n* = 3. **p < 0.01. Asterisk versus control+NC mimic. ^##^*p* < 0.01. Hash versus FEZF1-AS1+NC mimic

Starbase (http://starbase.sysu.edu.cn/index.php)predicted that PAX6 could be targeted by miR-363-3p (Fig. 4a). The luciferase activity of PAX6-wt was changed by miR-363-3p mimics. However, the luciferase activity of PAX6-mut was not affected (Fig. 4b). Western blot analysis revealed that FEZF1-AS1 overexpression contributed to PAX6 expression. The cells co-transfected with FEZF1-AS1 overexpression and miR-363-3p mimics reduced PAX6 expression. Conversely, FEZF1-AS1 silencing exhibited an opposite effect on PAX6 expression (Fig. 4c). The data indicated that FEZF1-AS1 affected miR-363-3p/PAX6 expression in retinoblastoma cells.

### The Effect of FEZF1-AS1 on Retinoblastoma Cell Viability and Apoptosis Through PAX6

**Fig. 5:**
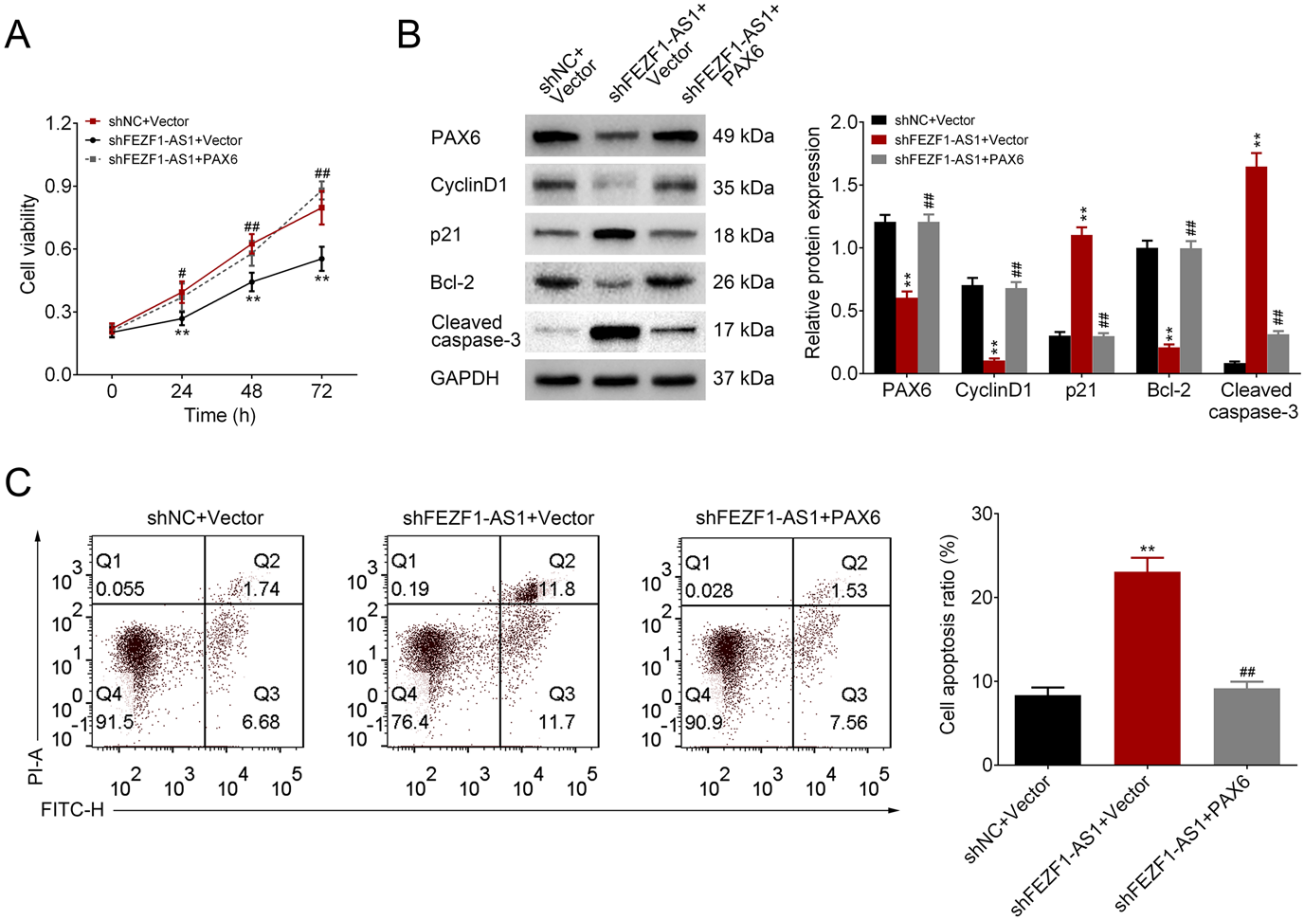
The effect of FEZF1-AS1 on retinoblastoma cell viability and apoptosis through PAX6. **a** CCK-8 was used to detect cell viability. *n* = 3. **p < 0.01. **b** PAX6, cyclinD1, p21, Bcl-2, and cleaved caspase-3 protein levels were examined using Western blot analysis. **c** Flow cytometry was used to measure cell apoptosis. *n* = 3. **p < 0.01. Asterisk versus shNC+Vector. ^##^*p* < 0.01. Hash versus shFEZF1-AS1+Vector

We then measured the effect of FEZF1-AS1 on retinoblastoma cell viability and apoptosis by PAX6. The results of CCK-8 assay revealed that FEZF1-AS1 silencing repressed cell viability, whereas PAX6 overexpression rescued the cell viability (Fig. 5a). Western blot analysis verified that a down-regulation of PAX6, CyclinD1, and Bcl-2 protein levels was caused by FEZF1-AS1 silencing, whereas an up-regulation of p21 and Cleaved caspase-3 protein levels was induced by FEZF1-AS1 silencing. However, the PAX6 overexpression had opposite effects on FEZF1-AS1 silencing-caused protein levels (Fig. 5b). Additionally, flow cytometry analysis proved that FEZF1-AS1 silencing promoted cell apoptosis, whereas PAX6 overexpression attenuated cell apoptosis (Fig. 5c). These results implied that FEZF1-AS1 promoted retinoblastoma cell viability and inhibited apoptosis through PAX6.

### *FEZF1-AS1 Knockdown Impaired Tumor Formation *In Vivo

**Fig. 6:**
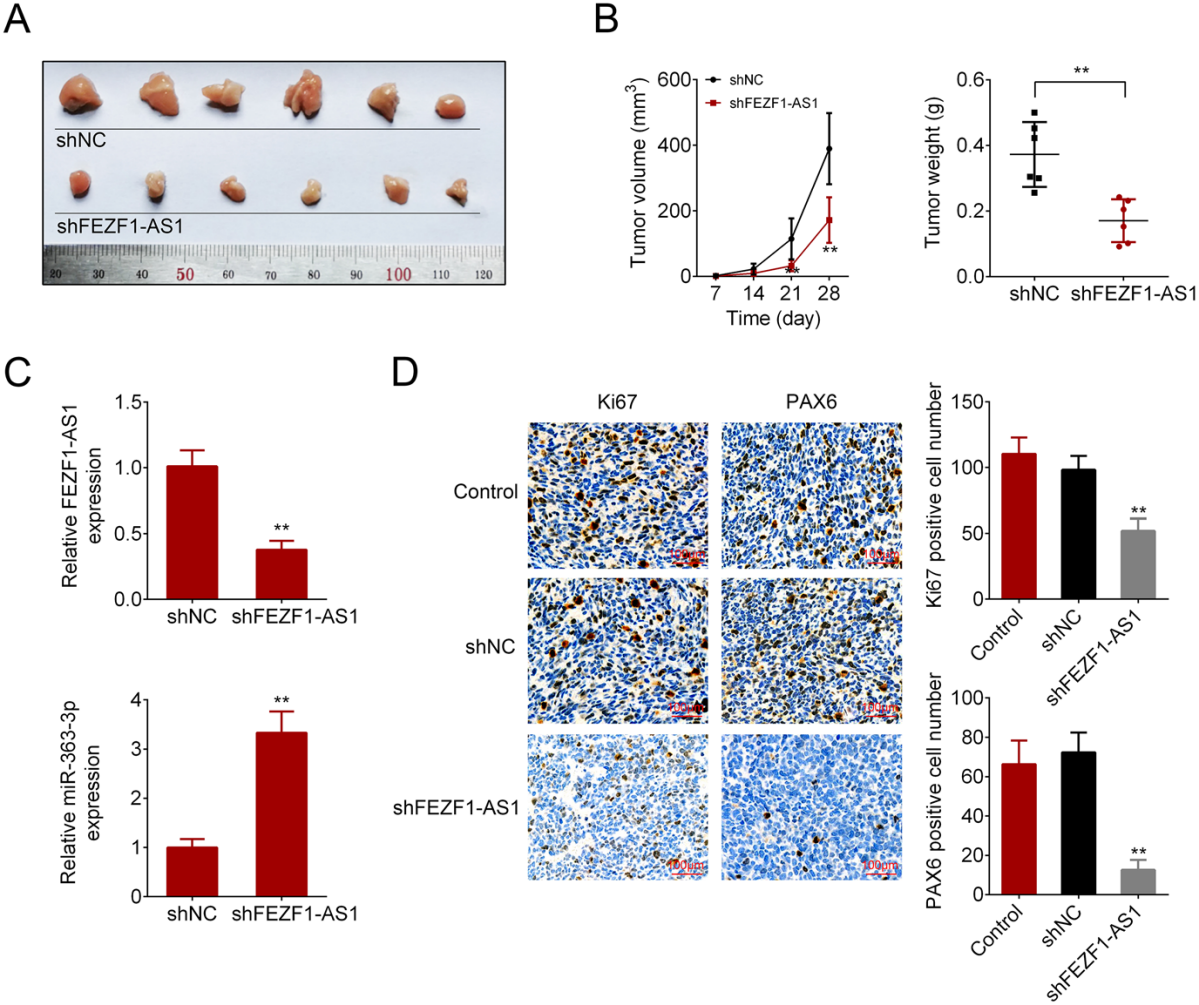
FEZF1-AS1 knockdown impaired tumor formation in vivo. **a** Tumor tissues were photographed under a microscope. **b** Tumor volume and weight were calculated. **c** FEZF1-AS1 and miR-363-3p expressions were confirmed via qRT-PCR. **d** Immunohistochemistry detected the Ki67 and PAX6 expression. *n* = 3. **p < 0.01

The effect of FEZF1-AS1 on tumor formation was evaluated via tumor xenograft experiment in vivo. At 28 days post-injection, mice were euthanized, and the tumors were excised and photographed (Fig. 6a). As shown in Fig. 6b, FEZF1-AS1 knockdown inhibited tumor volume and weight. The results from qRT-PCR revealed that FEZF1-AS1 knockdown down-regulated FEZF1-AS1 expression and up-regulated miR-363-3p expression (Fig. 6c). Immunohistochemistry demonstrated that FEZF1-AS1 knockdown suppressed Ki67, which was the marker of proliferation, and PAX6 expression (Fig. 6d). The data indicated that FEZF1-AS1 knockdown impaired tumor formation in vivo.

## Discussion

Here, we identified that FEZF1-AS1 was up-regulated in retinoblastoma tissues and cells. FEZF1-AS1 overexpression stimulated cell viability, cycle, and repressed apoptosis. FEZF1-AS1 silencing exhibited opposite effects. FEZF1-AS1 could sponge miR-363-3pand FEZF1-AS1 suppressed miR-363-3p level in retinoblastoma cells. Moreover, miR-363-3p was identified to bind to PAX6. FEZF1-AS1 promoted growth and inhibited apoptosis by regulating miR-363-3p and PAX6 in retinoblastoma.

Previous studies have shown that abnormal lncRNA expression was associated with progression of retinoblastoma. For example, lncRNA THOR was increased in the retinoblastoma patients and cells, enhanced cell growth, and suppressed cell apoptosis (Shang 2018). lncRNAUCA1 was up-regulated in retinoblastoma tissues and cells and elevated retinoblastoma cell proliferation and multidrug resistance (Yang et al. 2020). Interestingly, in our research, we demonstrated that FEZF1-AS1 was enhanced in retinoblastoma patients and cells. FEZF1-AS1 silencing inhibited cell viability in vivo and in vitro. Consistently, Quan et al. discovered that FEZF1-AS1 promoted proliferation in retinoblastoma (Quan and Wang 2019). Additionally, we found that FEZF1-AS1 could function in cell cycle and apoptosis. FEZF1-AS1 decreased the cell population in the G1 phase and increased cell population in the S phase. A decrease of cell apoptosis was caused by FEZF1-AS1 overexpression in retinoblastoma. We further proved that FEZF1-AS1 contributed to growth and inhibited apoptosis in retinoblastoma.

Increasing evidences have revealed that FEZF1-AS1 could compete endogenous RNA (ceRNA) to bind to miRNAs and regulate tumorigenesis and progression. For instance, Ye et al. proved that FEZF1-AS1 promoted progression of pancreatic ductal adenocarcinoma through miR-107 (Ye et al. 2018). Li et al. discovered that FEZF1-AS1stimulated cell growth by miR-610 in multiple myeloma (Li et al. 2018a). FEZF1-AS1 was found to exert oncogenic effects in retinoblastoma (Quan and Wang 2019). Thus, We hypothesize that FEZF1-AS1 may participate in regulation of cell growth in retinoblastoma via sponging miRNA. Interestingly, we confirmed that miR-363-3p could targetFEZF1-AS1. In addition, FEZF1-AS1 was verified to reduce miR-363-3p level in vivo and in vitro. The data indicated that FEZF1-AS1 may regulate cell progression in retinoblastoma through sponging miR-363-3p. Reportedly, miR-363-3p was involved in the cell development in diseases via targeting mRNA. For example, miR-363-3p was proved to repress cell proliferation and invasion in osteosarcoma through targeting SOX4 (Wang et al. 2019). Additionally, miR-363-3p took part in the inhibition of tumor growth and metastasis in colorectal cancer by SphK2 (Dong et al. 2018). As exhibited in our results, we identified the relationship between miR-363-3p and PAX6. Further, our study showed that FEZF1-AS1 silencing suppressed cell viability and cycle and promoted cell apoptosis. However, PAX6 overexpression exhibited opposite effects on FEZF1-AS1 silencing-caused cell viability, cycle and apoptosis. Notably, PAX6 was found to regulate the cell growth in retinoblastoma. For instance, Liu et al. revealed that miR-129-5p was demonstrated to inhibit cell progression in retinoblastoma through targeting PAX6 (Liu et al. 2019). Li et al. verified that miR-433 suppressed retinoblastoma cell proliferation and metastasis through direct targeting of PAX6 (Li et al. 2016). These findings indicated that FEZF1-AS1 participated in regulation of cell progression in retinoblastoma through miR-363-3p/PAX6.

However, accumulating evidences have shown that more lncRNAs regulated tumor progression by sponging miR-363-3p (Xie et al. 2019; Wang et al. 2020). Moreover, the miR-363-3p inhibitor/mimic and FEZF1-AS1 overexpression/knockdown should be reconfirmed in more retinoblastoma cell lines. In addition, the application of FEZF1-AS1 is not evaluated in clinical sample. Therefore, more experiments are still needed to be performed in the near future.

In conclusion, up-regulation of FEZF1-AS1 was demonstrated in retinoblastoma tissues and cells. FEZF1-AS1 overexpression facilitated to cell viability, cycle, and inhibited apoptosis. FEZF1-AS1 could sponge miR-363-3p and miR-363-3p-targeted PAX6. Furthermore, PAX6 overexpression alleviated the influence of FEZF1-AS1 on cell viability and apoptosis. The data implied that FEZF1-AS1 regulated cell progression in retinoblastoma via miR-363-3p/PAX6, which provides a candidate therapeutic target for retinoblastoma.

## Data Availability

All data generated or analyzed during this study are included in this published article.
